# Glucosinolates: Natural Occurrence, Biosynthesis, Accessibility, Isolation, Structures, and Biological Activities

**DOI:** 10.3390/molecules25194537

**Published:** 2020-10-03

**Authors:** V. P. Thinh Nguyen, Jon Stewart, Michel Lopez, Irina Ioannou, Florent Allais

**Affiliations:** 1 URD Agro-Biotechnologies Industrielles (ABI), CEBB (Centre Européen de Biotechnologie et de Bioéconomie), AgroParisTech, 51110 Pomacle, France; nguye050@chem.ufl.edu (V.P.T.N.); michel.lopez@agroparistech.fr (M.L.); irina.ioannou@agroparistech.fr (I.I.); 2 Department of Chemistry, University of Florida, Gainesville, FL 326011, USA; jds2@chem.ufl.edu

**Keywords:** glucosinolates, myrosinases, *Brassicaceae* family, *Moringacea* family, *Brassicales*

## Abstract

Glucosinolates (GSLs) are secondary plant metabolites abundantly found in plant order *Brassicales*. GSLs are constituted by an *S*-β-d-glucopyrano unit anomerically connected to *O*-sulfated (*Z*)-thiohydroximate moiety. The side-chain of the *O*-sulfate thiohydroximate moiety, which is derived from a different amino acid, contributes to the diversity of natural GSL, with more than 130 structures identified and validated to this day. Both the structural diversity of GSL and their biological implication in plants have been biochemically studied. Although chemical syntheses of GSL have been devised to give access to these secondary metabolites, direct extraction from biomass remains the conventional method to isolate natural GSL. While intact GSLs are biologically inactive, various products, including isothiocyanates, nitriles, epithionitriles, and cyanides obtained through their hydrolysis of GSLs, exhibit many different biological activities, among which several therapeutic benefits have been suggested. This article reviews natural occurrence, accessibility via chemical, synthetic biochemical pathways of GSL, and the current methodology of extraction, purification, and characterization. Structural information, including the most recent classification of GSL, and their stability and storage conditions will also be discussed. The biological perspective will also be explored to demonstrate the importance of these prominent metabolites.

## 1. Introduction

**Figure 1:**
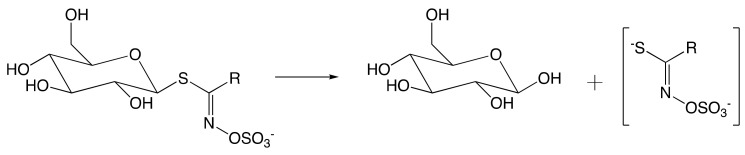
Hydrolysis of glucosinolate (GSL) by myrosinase (MYR) upon tissue disruption. (R = alkyl, aryl, indole).

Amino acid-derived glucosinolates (GSLs), which are secondary plant metabolites constituted of a sulfate and thioglucose moiety, play important biological roles in the *Brassicaceae* family defense system, crops of great relevance to agriculture [1]. The coexistent thioglucosidase myrosinase (MYR) (EC 3.2.1.147) originally segregated within plants [2], will come in contact with GSL upon tissue disruption. Consequently, the enzymatic hydrolysis of GSL occurs to form glucose, and an unstable aglucone that undergoes degradation to afford a wide range of active components in response to environmental stresses (Figure 1). Along with the aforementioned role in the defense system, GSLs are likely involved in the survival system of the *Brassicaceae* family. In a study on *Arabidopsis thaliana* under abiotic stress (e.g., high salt), the overproduction of short-chain aliphatic GSL and underproduction of indolic GSL in leaves occurred [3], suggesting the adaptation of the plant in response to environmental stresses, and thus demonstrating the biological importance of GSLs in the *Brassicaceae* survival system, besides their prominent role involved in defense mechanism.

With over 130 GSL structures have been discovered and validated to date [4], variable side-chains in GSL structures (R group in Figure 1) suggest their implication in different biological activities. Whereas sinigrin, the most abundant aliphatic GSL in *Brassicaceae*, is directly implicated in controlling soil-borne plant pests [5], indolic GSLs are likely involved in insect-deterring functions [6]. Taken together, biosynthetic pathways and regulation of different groups of GSLs will, therefore, lead to an understanding of the structural diversity of GSLs.

Synthetical approaches to GSLs have been devised with regards to their involvement in different biological processes in plants as well as their health benefits (reviewed in [7,8]). Several natural GSL such as sinigrin [9], glucobrassicin [10], along with a number of artificial GSLs [11] have been obtained. These syntheses appear to be straightforward and easily accessible. Nevertheless, the stereoselectivity challenge prompted by certain natural GSL remains to be circumvented [12]. As a result, the isolation of natural GSL from biomass is the method of choice to complement the limitation of synthetical approaches.

As natural GSLs are water-soluble components [8], the extraction of these secondary metabolites from various biomass types is achievable via a simple maceration [13]. The effect of the extraction process onto their biological activities, the stability, and the concentration of extracted GSLs are critical factors for determining the adequation of the isolation method. We, therefore, suggest that the stability and storage method of GSLs must be included in the extraction process in order to provide a thorough insight into the selected extraction strategy. For instance, it is often mentioned that high temperature prompts the degradation of GSLs [14,15]. Therefore, extraction at a lower temperature will allow the full recovery of GSLs without altering their structure and corresponding biological activities.

The characterization of GSLs has been well developed [16]. Extracted GSLs undergo purification by liquid chromatography followed by characterization either by mass spectrometry or UV absorption. Additionally, Nuclear Magnetic Resonance (NMR) spectrometry analysis is often performed to confirm the structure of GSLs. In the case of GSLs extracted from a complex matrix, an extra desulfation step using sulfatase is needed to yield the corresponding desulfated GSLs (desGSLs) prior to the characterization step. Although considered as robust, the analysis of desGSLs is time-consuming as the incubation of sulfatases with GSL requires approximately 24 h prior to the characterization.

Classification of characterized GSL structures has also been well studied. Several classification systems have been proposed based on the chemical structure of the GSL precursors, such as the distinctions between “aliphatic”, “aromatic”, and “indole” [17,18], and, the most recent criterion suggested by Blaževic et al., the presence or absence of aromatic motifs [4].

GSLs are omnipresent in *Brassicaceae* plants and their processed products [19]. Upon assimilation, both positive and negative effects of GSLs remaining in processed products have been probed in animal nutrition [19,20]. For human nutrition, the negative effects of GSLs remain to be elucidated due to the low abundance of literature evidence. However, the health benefits of consuming vegetables containing GSLs (e.g., broccoli, cabbage, and many more) are often mentioned, including antibacterial, anticancer, antioxidant, and anti-inflammatory functions [8,21].

The aim of this review is to provide an overview of the chemical and biochemical aspects of GSLs. Biosynthesis and current chemical synthetic strategies of GSLs will also be discussed. The current extraction strategy, along with the purification and characterization methods of GSLs will also be explored to complement the limitations of chemical syntheses. Also, we will include in this review, the most recent classification of GSLs by their side-chain structures proposed by Blaževic et al. [4]. Additional scopes concerning the stability of extracted GSL and isolation methods will also be explored to emphasize the potential use of these natural molecules as bioactive compounds. The detailed mechanism of MYR-mediated hydrolysis, the fate of the GSL aglucone, and its health benefits will furthermore be developed in Section 7 of this review.

## 2. Natural Occurrence of Glucosinolates

The abundant presence in *Brassicaceae* vegetables and condiments makes GSLs of interest to human society. To date, the therapeutic benefits of GSLs [8] have drawn more attention to this class of secondary metabolites, alongside with their original food purposes. Although several synthetic approaches have been documented [11], most natural GSLs reside in plants, with more than 130 different GSLs having been validated [4,18].

GSL concentration is unequally distributed throughout the plant body. For instance, in *Brassica napus*, the GSL concentration in the seed is greater than that in leaves [22]. This variation appears to be more relevant in root vegetable crops (*Moringacea* family) than that in oilseed crops (*Brassicaceae* family). Moreover, the GSL profile varies depending on the tissue type. Although aliphatic GSLs predominate both in leaves and in seeds, indole GSLs are more abundant in leaves than in seeds [23]. This difference may be related to different functions of different parts of plants. A study of Troufflard et al. [24] showing that *A. thaliana* accumulated more GSL in the roots than in the shoots in response to abiotic stress is clear evidence to support the last suggestion. For further literature on plant response to abiotic stress involving GSL accumulation, we recommend the review by Martínez-Ballesta et al. [25].

Breeding approaches are often employed to obtain crops with low GSL content for food or feed purposes [26,27,28], while those with high GSL content remain of interest for non-food applications. Therefore, the choice of species should be carefully considered with regard to the downstream purposes of raw materials. We also suggest that growth conditions should be highly regarded in order to adapt the chosen crops to their cultivating environment.

The occurrence of GSL varies among different species within the same order, as shown in Table 1. These variations even occur for the same crop depending on the years. For instance, Ishida et al. reported that the amount of GSLs in the same crops of Japanese radish varied between 2005 and 2009 [29]. It is assumed that the accumulation of GSLs within plants highly depends on environmental factors such as the weather that undergoes slight changes through the years, thus directly impacting the GSL contents of the crops. Therefore, the GSL content of the same crops must be kept updated annually, or more frequently if needed.

## 3. Glucosinolates: Biosynthetic and Chemical Synthetic Pathways

### 3.1. Biosynthesis of Glucosinolates in Plants

**Figure 2:**
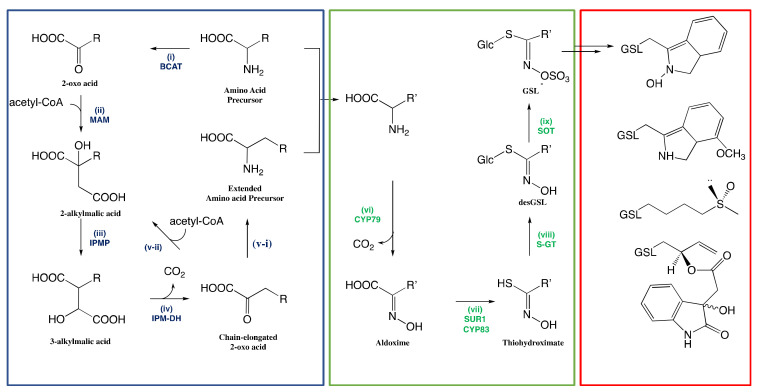
Three separate phases of glucosinolate biosynthesis: R indicates the variable amino acid precursors, and R’ indicates either original or extended amino acid. The blue box indicates the chain elongation phase, the green box indicates the reconfiguration phase yielding the core structure of glucosinolate, and the red box indicates the glucosinolate side-chain modification phase of the glucosinolate core structure with some examples from Table 2. The figure was adapted from the biosynthesis of GSL proposed by Graser et al. [46].

The biosynthesis of GSLs in plants has been studied extensively [44,45]. As depicted in Figure 2, this pathway is composed of three separate phases: (1) chain elongation that consists of the insertion of a methylene group into the side-chain of aliphatic amino acids, (2) metabolic reconfiguration of the amino acid moiety to afford the core structure of GSL, and (3) the modification of the core structure to yield GSL bearing various aglucone structures.

#### 3.1.1. Side-Chain Elongation of Amino Acid

In an early study of GSL biosynthesis in the 1960s, Chrisholm and Wetter used radio-labeled methionine as an aglucone precursor to provide the first evidence for the side-chain elongation phase [128]. More recent studies by Graser et al. confirmed the chain elongation existence by characterizing the extension of 2-oxo acid using radioisotope and tandem mass spectrometry with stable isotope coupling analysis [46,129].

The chain elongation phase is initiated with the deamination by branched-chain amino acid aminotransferase (BCAT) that transforms the parent amino acids into the corresponding 2-oxo acids (Step (i), Figure 2). The next stage consists in a three-step transformation cycle where (1) the resulting 2-oxo acid is condensed with acetyl-CoA by a methylthioalkylmalate synthase (MAM) to form a 2-malate derivative (Step (ii), Figure 2), (2) 2-malate is then isomerized to 3-malate derivative by an isopropylmalate isomerase (IPMP) (Step (iii), Figure 2) followed by (3) a decarboxylation by an isopropylmalatedehydrogenase (IPM-DH) to yield an intermediate elongated 2-oxo acid (Step (iv), Figure 2) [130]. This intermediate can either undergo a transamination to provide extended amino acid for the next phase (Step (v–i)); or reenter into the transformation cycle for further elongation (Step (v–ii), Figure 2) [17].

#### 3.1.2. Reconfiguration of Amino Acid to Glucosinolate Core

##### The Conversion of Amino Acid to Aldoximes

The reconfiguration begins with the oxidation of the amino acid into corresponding aldoximes (Step (vi), Figure 2). The oxidation is catalyzed by three different enzyme systems: cytochrome-P450 (CYP79) dependent monooxygenase, flavin-containing monooxygenase, and peroxidase [44]. The involvement of each enzyme system depends on the nature of amino acid precursors [1]: GSL from tyrosine or phenylalanine precursors, and homophenylalanine or elongated methionine GSLs are catalyzed by cytochrome-P450 dependent and independent monooxygenases, respectively; while plasma membrane-bound peroxidases produce GSL from tryptophan.

##### The Conversion of Aldoximes to Thiohydroximic Acids

**Figure 3:**
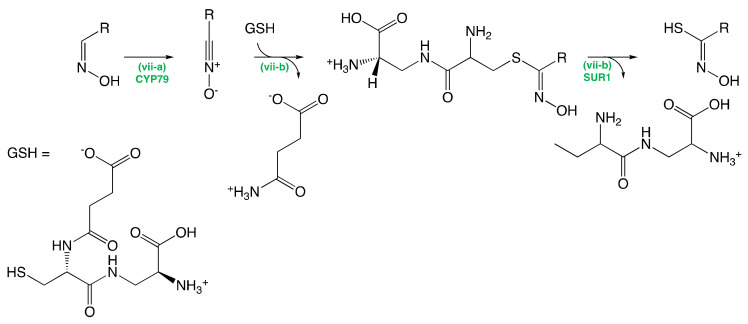
Conversion of Aldoximes to Thiohydroximic Acids. GSH: Glutathione.

CYP83 cytochrome monooxygenases activate the aldoxime resulting from the oxidation of the amino acid to give the corresponding thiohydroxymate (Step (vii-a), Figure 3). The activated aldoxime is then conjugated to glutathione (GSH), which acts as a sulfur-donor [6] to yield the corresponding thiohydroximate intermediate (Step (vii-b), Figure 3). The newly formed S-alkyl-thiohydroximate intermediate is then cleaved by a C-S lyase: SUR1 to provide the corresponding thiohydroximates [131] (Step (vii-c), Figure 3). An experiment conducted by Czerniawski and Bednarker while studying the biosynthesis of indolic GSL showed the formation of intermediate GSH-conjugates that suggests the involvement of GSH within the biosynthesis of GSLs [132].

##### The Formation of Glucosinolate Core

Thiohydroximates follow a subsequent transformation catalyzed by UDP-glucose:thiohydroximic acid *S*-glucosyltransferases (S-GT) (Step (viii), Figure 2), and desulfoglucosinolate sulfotransferases to afford GSL core structure with corresponding side-chains (Step (ix), Figure 2). Transferases involved in the formation of GSL core have been identified and reported in the literature [133,134]. This identification clarifies how GSL core has been formed via the transfer of glucose and sulfate moieties by corresponding transferases.

#### 3.1.3. Natural Side-Chain Modification of Glucosinolates

Side-chain modifications of newly formed GSL core structures are frequently mentioned [4,135]. Chemical transformations of GSL side-chains occur in vivo via enzyme-catalyzed oxidations, eliminations, alkylations, and esterifications [136]. Most reported side-chain modifications are related to methionine-derived GSL [137].

These side-chain decorations increasingly draw interests in regard to their influence on the direction of myrosinate-catalyzed hydrolysis as well as the resulting activities of hydrolysis products [45]. Moreover, these modifications contribute to the structural diversity of this class of molecules. As a result of the diversity of these side-chains, a number of GSLs with more complex side-chains results in multiple biological activities in plants [130].

#### 3.1.4. Regulation of Glucosinolate Biosynthesis

Studies of the regulatory system in the plant model *Arabidopsis thaliana* that employs a genetic approach combined with structure profiling provide further information about the regulation of GSL biosynthesis in *Brassica* plants. Quantitative Trait Locus (QTL) is a region of DNA (Deoxyribonucleic acid) that influences a quantitative phenotype trait [138]. Analyzing the expression of phenotype traits, GSL, in this case, in *A. thaliana* allows identifying new functional loci [130]. *GS-ELONG, GS-OX, GS-AOP*, and *GS-OH* have been identified to be responsible for side-chain variability of aliphatic GSL [139]. In the case of indolic GSL, QTL mapping has been combined with transcript profiling and subsequent *cis*-expression QTL to prompt the regulation gene of 4-methoxy indolyl-3-methyl GSL in *A. thaliana* [140]. Wentzell et al. have successfully identified a locus that regulates the expression of aliphatic and indole GSLs by mapping the expression QTL with the expression of phenotype traits in *A. thaliana* [141]. For further genomic insights, a review of Sønderby et al. is highly recommended [130].

The regulatory system of GSL biosynthesis in plants is complex. In spite of the extensive studies on GSL biosynthesis [142,143,144], the genetic and biochemical nature of their regulation remains to be elucidated. Further investigation should be conducted to examine the biosynthesis of GSL, which can lead to a deeper understanding of the biological role of GSLs under environmental stresses as the regulation of these metabolites is tightly related to the survival of the plants.

### 3.2. Chemical Synthesis of Glucosinolates

**Figure 4:**
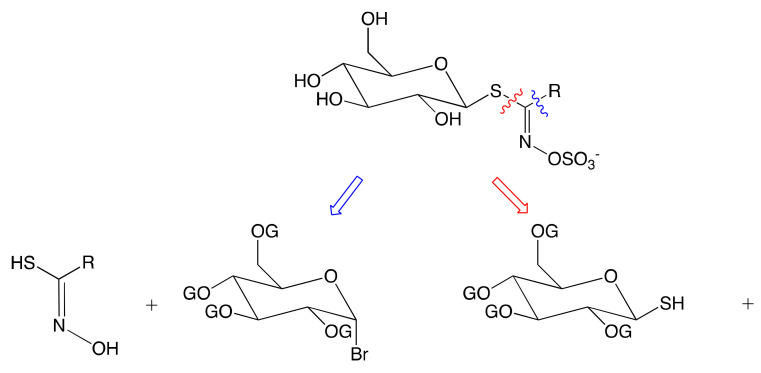
Retrosynthesis approach to GSL synthesis: anomeric disconnection (blue), hydroximate disconnection (red). OG: suitable protecting group.

The synthetic chemical approach is an efficient way to produce pure, naturally occurring, and artificial GSL. Two synthetic strategies have been proposed based on the disconnection between glucose and aglucone moieties: anomeric disconnection and hydroximate disconnection (Figure 4) [11].

#### 3.2.1. Anomeric Disconnection

**Figure 5:**
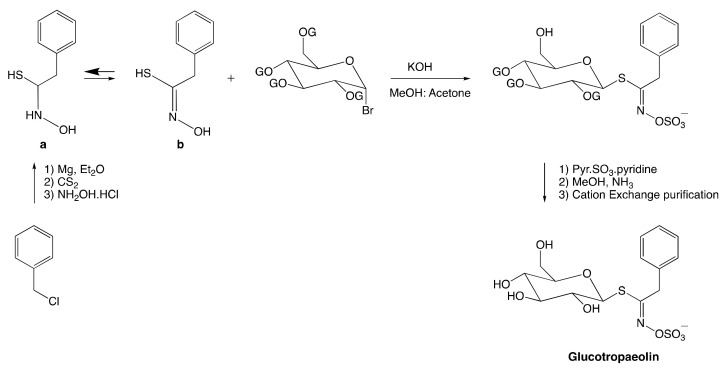
Synthesis of glucotropaeolin. OG represents a suitable protecting group.

The anomeric disconnection involves a standard electrophilic glucosyl donor and a thiohydroxamic acceptor. The method was established by Ettlinger and Ludden [145]. Figure 5 illustrates the synthesis of glucotropaeolin following this method.

The synthesis starts with the addition of benzyl magnesium chloride to carbon disulfide. The reaction was then treated with aqueous hydroxylamine hydrochloride at 0 °C to form in situ the nucleophile **a**, in equilibrium with the desired nucleophile **b** (ca. 33%) (Figure 5). The latter is then reacted with protected *α*-bromoglucose under basic conditions to provide the corresponding glucosyl thiohydroximate. The next step relies on reacting glucosyl thiohydroximate with sulfur trioxide pyridine. The resulting peracetylated glucotropaeolate anion is then crystallized with either potassium or tetramethylammonium salt. Ultimately, glucotropaeolin undergoes purification by cation exchange chromatography. This method is believed to result in low-yield due to the formation of unstable alkylthiohydroxamic intermediate. Furthermore, moderate efficiency of the nucleophilic displacement at the anomeric position of the glucosyl halide counterpart is another reason that contributes to the unpopularity of this approach. To the best of our knowledge, this synthetic procedure has never been optimized further since [11]. Therefore, most of GSL synthetic pathways rather follow the hydroximate disconnection.

#### 3.2.2. Hydroximate Disconnection

As outlined above, this methodology involving a 1,3-addition of a protected thio-β-d-glucopyranose on a highly labile nitrile oxide is the most popular methodology in GSL synthesis. However, nitrile oxide has to be generated in situ from the corresponding hydroximoyl precursor [4]. Multiple approaches have been developed to access to this labile precursor from aldoximes, aliphatic nitronates, and nitrovinyl derivatives.

##### The Aldoxime Pathway

**Figure 6:**
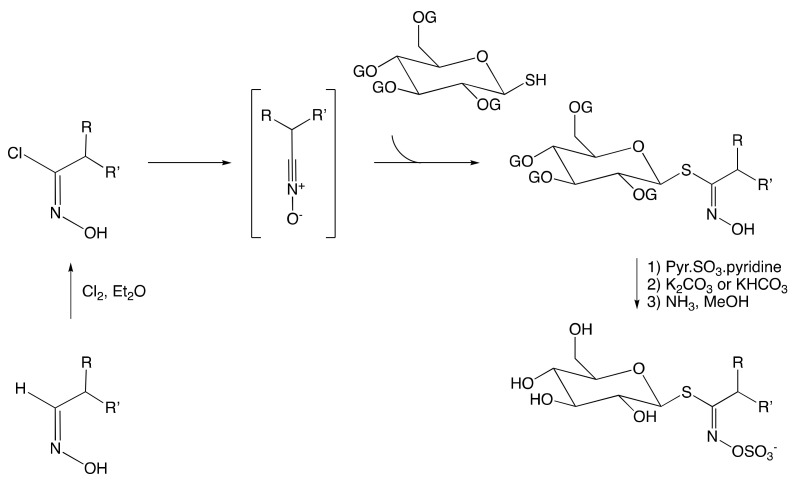
Synthesis of GSL following the aldoxime pathway (R, R’ = H, alkyl, or aryl).

In the early 1960s, M.H. Benn devised the first synthesis of GSL that employs the aldoxime pathway (Figure 6) [146]. The hydroxamic chloride was prepared by chlorination of the precursor aldoxime and reacted in situ with a base to yield the unstable corresponding nitrile oxide. The latter was then reacted with protected glucosyl thiol to give the corresponding glucosylthiohydroxamate that, upon subsequent sulfonation, affords the desired protected GSL. The final step was the deprotection to provide the desired glucosinolate under its salt form. Although the aldoxime pathway is a method of choice to synthesize GSLs, the halogenation tolerance of side-chain aldoxime remains a limitation of this method [11]. As a result, alternative approaches employing nitronate and nitrovinyl pathways have been investigated.

##### The Nitronate and Nitrovinyl Pathway

**Figure 7:**
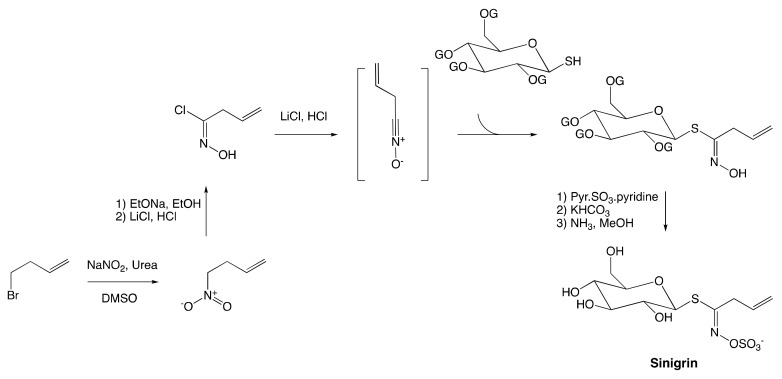
Synthesis of sinigrin employing nitronate pathway.

In the previous methodology, the low tolerance of many vinyl and aryl aldoxime precursors toward the halogenation step was proven a critical issue. To overcome this limitation, an alternative method to generate the key hydroximoyl chloride intermediate via the formation of nitronate was established for the first time by Benn and Ettlinger [9]. In their study, sinigrin was successfully synthesized from but-3-enyl bromide employing the nitronate pathway (Figure 7).

The synthesis starts with the conversion of but-3-enyl to the corresponding nitronate anion. The medium containing newly formed nitronate is chilled down to 0 °C, and lithium chloride-hydrochloric is added to yield the corresponding but-3-enohydroxamoyl. The latter then undergoes the same established pathway as that of aldoxime by reacting with tetraacetyl-β-d-glucopyranosyl mercaptan to afford the corresponding thiohydroximic acid, which is then transformed to desired sinigrin as depicted in the last step of Figure 7.

**Figure 8:**
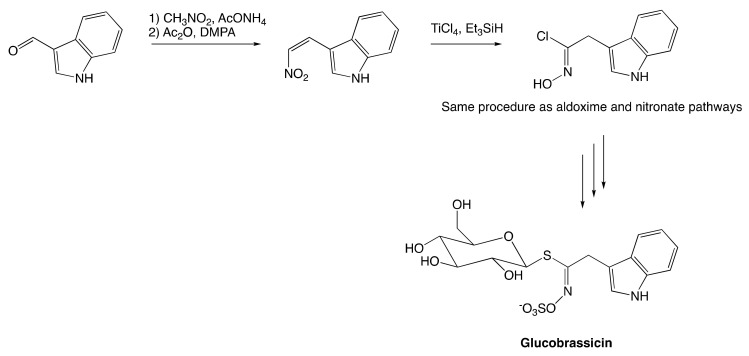
Synthesis of glucobrassicin.

The nitrovinyl pathway development owes to the discovery of one-step conversion of nitroalkenes to hydroximoyl chlorides [147]. As depicted in Figure 8 for the synthesis of indole GSL, glucobrassicin, the conversion of nitroalkenes relies on the reaction of nitrovinyl derivatives with triethylsilane (i.e., hydride source) in the presence of a Lewis acid to provide substituted acetylhydroximol chlorides [10]. This hydroximoyl intermediate then follows the same pathway as aldoxime and nitronate pathway to afford the desired GSL.

Many successful syntheses of vinyl, aryl, and indole GSL through the nitronate and nitrovinyl pathway have been performed and reported [4,11]. However, one exception [148] has shown that the nitronate pathway is less efficient than the aldoxime one for the synthesis of aryl GSL. Moreover, the lack of stereoselectivity in the previously established pathway is revealed [11,12]. Based on these considerations, higher stereoselective synthetic methodologies remain to be designed to tackle these challenges.

## 4. Extraction, Purification, and Characterization of Glucosinolates

### 4.1. Extraction of Glucosinolates

Glucosinolates are water-soluble components with a very low octanol-water partition coefficient owing to their ionized sulfate and hydrophilic thioglucose moieties [8]. Therefore, the extraction of these metabolites from plant materials mainly relies on solid-liquid extraction with boiling water [13,149] or aqueous organic solvent as an extraction solvent [150,151,152,153].

Various modifications of extraction parameters, including solvent composition, extraction temperatures, and tissue disruption, have been investigated in order to optimize the extraction process. Doheny–Adams et al. studied the effects of these parameters on the extraction of GSLs from several *Brassica* plants [83]. Different extraction conditions, where boiled water and mixture of methanol/water were used as extraction solvents, have been studied. Tissue disruption prior to GSL extractions was also carefully investigated. As a result, the use of a freeze drier for tissue disruption is unnecessary for short term storage of plant tissue samples. Freeze drying, in contrast, is advised for long term storage in order to maintain the GSL recovery yield of the established process. Use of a cold mixture of 80/20 methanol/water as an extraction solvent instead of a boiling mixture of 70/30 methanol/water appears to be advantageous for industrial scale due to the reduction in the number of steps in the process while being less hazardous with an improved or comparable GSL recovery rate.

Originally established by Thies [154], isolation of intact GSL extraction has been recently improved by Förster et al. [40]. The strategy started by extracting GSL from *Moringa oelifera* leaves with 70% methanol at 80 °C. The extract was then purified by chromatography to yield a purified GSL fraction. Additional recrystallization steps are needed in order to yield the final pure GSL mixture. This method was reported to yield up to 600 µmol of GSL per gram of dry material. With regards to the GSL amount isolated from *M. oleifera* leaves, this strategy appears to be efficient and accessible. On the other hand, employing a hot extraction with methanol might lead to the partial degradation of GSLs, which can be proved by the observation of the formation of artifact GSLs and loss of the acetylated GSL, as observed by the authors. Therefore, the extraction at high temperatures should be carefully considered concerning the degradation of GSL. Besides the intact GSL extraction, Förster et al. also employed a desulfation strategy in order to isolate GSL from *M. oleifera* leaves [41]. In this strategy, the extraction step followed the same protocol as that of intact GSL. Rather than being directly eluted, bound GSLs were treated with a cleaned-up *Helix pomatia* sulfatase solution in order to remove the sulfate group of GSL. After overnight incubation, the desulfated GSL was easily eluted by flushing the column with ultra-pure water. Despite the qualitative difference between the desulfation and the intact extraction, the resulting total amount of GSL yielded by both methods was reported to be similar. Nevertheless, the formation of artifacts and loss of acetylated GSL observed while employing the desulfation approach for isolating *M. oleifera*’s GSL advised that the desulfation method is not appropriate to recover GSLs from plant materials.

The use of physical accelerators to intensify the extraction, such as ultrasound, has been developed to enhance the extraction yield [155]. This methodology consists of the application of ultrasound during the extraction step, which improved the GSL recovery rate and time efficiency, as well as reducing the amount of extraction solvent. Taken together, extraction productivity has been significantly increased over the conventional extraction method, as it prompts to the elimination of outer pectinous materials under ultrasound treatment, which facilitates the recovery of GSLs from plant materials.

Supercritical carbon dioxide extraction (Sc-CO_2_) nowadays increasingly drawn more attention as an alternative and environmentally friendly technique for solvent extraction. The advantages of using Sc-CO_2_ for GSL extraction over conventional methods from *Eruca sativa* leaves have been recently reported [156]. The results showed that a mixture of Sc-CO_2_/water allowed efficient extraction of GSLs from the plant materials, with the recovery yield of GSL determined to be 64% of the total GSL amount. The recovery yield remained stable at the temperature ranged from 45 to 75 °C, with a constant pressure of 30 MPa. Moreover, the substrate selectivity of the extraction can also be controlled. Indeed, by increasing the pressure from 15 to 30 MPa, GSLs were selectively recovered over the polyphenols, which were more favorably extracted at lower pressure. Despite the lower extraction yield compared to the conventional solvent extraction using boiling water, Sc-CO_2_ selectively extracted GSL from other secondary metabolites while preserving bioactivities of extracted GSL.

Accelerated solvent extraction (ASE) is an extraction technique carried out under pressure and an inert atmosphere with a range of extraction temperatures from 35 to 200 °C. It has been proved that ASE quantitatively enhanced the recovery yields of hydrocarbons from reference materials [157]. This technique has been applied to recover GSL from *Isatis tinctoria* leaves with success [150]. The extraction conditions have been optimized and reported to be as following: raw material particle size: 0.5 mm, temperature: 50 °C, extraction solvent: 70% methanol in water, and three extraction cycles of 5 min. The recovery yield of the study has been reported to be over 97%. It is noted that the degradation of GSLs has been observed where temperature extraction exceeded 50 °C. This information has confirmed the thermal sensitivity of GSLs during the extraction process. ASE was also employed to extract GSL from *Lepidium sativum* [158]. In this study, ASE, however, did not show any relevant recovery yield advantage compared to the conventional maceration extraction technique. Moreover, GSLs recovered by ASE appeared to be less efficient in reducing the bacterial growth inhibition compared to those issued from other extraction techniques. This observation suggested that the partial degradation of extracted GSL occurred during the extraction process, which led to lower biological activities.

As GSLs are recovered along with other water-soluble components from the biomass such as proteins and phenolic compounds, a selective GSL extraction method is desired to efficiently isolate these metabolites. The conventional solvent extraction using aqueous-alcoholic solvent remains often used due to its simplicity, speed, and cost-efficiency, as well as a high recovery rate of GSLs. However, the extract obtained is often subject to successive purification process employing chromatography, which is time and cost consuming. Performing the extraction using advanced intensification techniques such as ultrasound accelerated extraction, Sc-CO_2_ extraction, or ASE has been found to be advantageous over the conventional method in terms of time, solvent consumption, and energetic efficiency. In addition, the selectivity of these extraction methods with respect to GSL is high and enables GSL to be isolated efficiently from other components of the biomass. However, these processes still have a fairly high cost to be commonly used in the industry.

### 4.2. Purification and Separation of Glucosinolates

Isolation of GSLs from the aqueous extract previously obtained appears to be particularly arduous as these metabolites are extremely hydrophilic. Early reported isolation of GSLs from rapeseed had been devised by Thies, where the isolation of sinigrin took advantage of the ionized nature of GSL [154]. The method consists of adsorbing targeted GSLs onto weak anion-exchange resin DEAE Sephadex A25. The bound GSLs were then eluted by adding a high concentration of potassium sulfate solution. The eluate was next concentrated under vacuum and then purified again using weak cation-exchange CM Sephadex C25. The eluate obtained from the second purification was subsequently concentrated and recrystallized to afford a pure solid GSL. Gram scale of sinigrin and glucotropaeolin with high purity have been isolated by employing this method. This purification approach was recently employed by Wang et al. [159]. The adsorption/desorption process of negatively charged GSL was performed on macroporous ion-exchange resins. The process was reported to successfully recover sinigrin, the main GSL in *Brassica juncea* L., at 58% of purity with a recovery rate near 80%.

Most of the documented current separation methods rely on chromatography techniques. This conventional method allows isolating the integrality of GSLs from plant materials. Charpentier et al. have separately isolated progoitrin and gluconapin with success using chromatography on an alumina column [160]. The recovery yields have been reported to be 96% and 98% from the aqueous extract for progoitrin and gluconapin, respectively. By employing preparative scale High-performance liquid chromatography (HPLC), Rochfort et al. have established an isolation process to isolate 17.6 mg of pure glucoraphanin from 3 g of broccoli seeds [103].

High-speed counter-current chromatography (HSCCC) is a hybrid technique that combines liquid chromatography and liquid-liquid counter-current distribution, in conjunction with the use of centrifugal force [161]. Fahey et al. have successfully separated different GSLs from broccoli seed extract employing this technique [162]. It is noteworthy that the partition coefficient of immiscible solvents is crucial in order to successfully separate similar GSL. The optimal solvent system was determined to be 1-propanol–acetonitrile–saturated aqueous ammonium sulfate–water, 1–0.5–1.2–1. The results showed that the separation of different GSLs in the extract was achieved with a high recovery rate (over 88% of the overall yield).

Although conventional techniques employing a liquid chromatography system enable the isolation of individual GSLs with high purity, these processes demand not only precise and high-cost instruments, operating systems, types of column, but also a large amount of high-salt and high-polar solvents with massive energy and time consumption. Ion-exchanger resin via batch adsorption, on the other hand, refers to a straightforward purification of total GSL with regards to its rapidity and ability to be performed at the industrial scale. However, this method does not allow a facile separation between different GSL residing in the extract. As a result, the selection of the purification method should be carefully considered concerning the downstream applications of isolated GSLs.

### 4.3. Characterization of Glucosinolates

Glucosinolates, once recovered and purified from plant materials, can be characterized. Their qualitative characterization is mainly conducted using liquid chromatography-tandem mass spectrometry ((U)HPLC-MS^n^) [4,96,160]. Nuclear magnetic resonance (NMR) spectrometry is often used as the ultimate confirmation allowing unambiguous determination of GSL structures [4,51]. Other characterization techniques have been used to complement the previous conventional analytical methods. Crystallization enables the visualization of glucoiberin [102] and sinigrin [163] by X-ray analysis. These are the only crystal structures of GSL that have been documented to our knowledge. Fourier-transform infrared spectroscopy analysis is often used to confirm the presence of sugar moiety, which is considered as a characteristic of these metabolites [49,96].

The Desulfation procedure is often employed to determine GSLs structure [83,151]. This method consists in the immobilization of intact GSL on an anion exchanger cartridge via the characteristic sulfate group. Applying *Helix pomatia* sulfatase directly on the anion exchanger cartridge allows removing the sulfate group localized on the aglucone moiety of bound GSL. DesGSLs are then released from anion ion resin and eluted by flushing the cartridge with ultra-pure water. Analysis of desGSL permits deducing the corresponding intact GSL structures.

Although the desulfation procedure enables distinguishing between different isomers of several GSLs [50], this is not a universal approach for characterization of GSLs as certain less stable desGSL leads to analytical difficulties [18]. Also, GSLs with a negatively charged side-chain cannot be characterized by the desulfation procedure as it is impossible to elute desGSL from an anion exchanger cartridge due to the negative charge of these latter side-chains [4]. Despite the aforementioned disadvantages, the identification of GSLs employing this method is recommended by present reviewers and remains the conventional method in GSL characterization [151].

The productivity of the whole extraction process relies on the correct identification of GSLs. Thus, extensive characterization should include at least ^1^H NMR, mass spectrometry (MS), and infra-red (IR) to satisfy prerequisite standards. Further spectroscopic proofs, such as ^13^C NMR, MS-MS, and elemental analysis, obviously adds valuable structural information on the GSL of interest. Although the desulfation procedure followed by conventional analysis remains the conventional method for GSL identification, this method still has limits regarding the stability of desulfated products and other problems involving the diversity of the GSL side-chain. Moreover, desulfation is time consuming despite of the robustness of the method. Therefore, further analytical methodology is required in order to expand the scientific understanding of these metabolites.

## 5. Structure and Classification of Glucosinolates

GSLs are anions composed of thiohydroxymates carrying an *S*-linked β-glucopyranosyl residue and an *N*-linked sulfate bearing an amino acid derived side-chain, which is referred to as the “R group” in the general structure Figure 1. This side-chain is subject to broad structural variation with associated biological functionalization associated [4].

GSLs are frequently classified in three main families based on the nature of these amino acids, namely “aliphatic”, “aromatic”, and “indole” [130]. However, that classification is thought to be of little biological and chemical significance, according to the recent review by Blaževic et al. [4]. The authors have then introduced a classification system based on amino acid precursors. In their review, they identify over 130 validated GSLs which were classified into nine panels from **A** to **I** depending on three main criteria: (1) amino acid precursor, (2) type of degradation product, either volatile or non-volatile isothiocyanates (ITC) or oxazolidine-2-thione; and, (3) presence and absence of an aromatic moiety in the GSL. Table 2 gives an example of how some GSLs are classified according to criteria proposed by Blaževic et al.

The proposed criteria offer a reliable system for GSL classification based on the chemical and biochemical properties of GSLs and their degradation product while conserving the information related to their amino acid precursor. The criterion concerning the presence or absence of an aromatic moiety in the GSL is meaningful as it allows the quick separation of a large amount of GSLs while using UV detectors. The usefulness of this criterion was demonstrated by the authors by separating GSLs of which Phe, Tyr, and Trp are precursors, from other non-aromatic groups. Moreover, further subgrouping within the aromatic group that separates indolic GSL from other phenylalkyl and less common aromatic GSLs appears to be of use.

## 6. Stability of Glucosinolates

### 6.1. Effects of Processing Methods on Glucosinolate Profile

Besides the chemical degradation involving MYR-catalyzed hydrolysis, the thermal degradation of GSLs is often mentioned [14,15,164]. As a result, GSL profiles of cooked *brassica* vegetables are altered at a different level depending on employed culinary techniques, such as cooking, steaming, and microwaving. The reduction of red cabbage (*Brassica oleracea*) indolic GSL during the cooking process was observed [165]. The content of glucobrassicin (Structure shown in Figure 8) and its homologs were drastically declined due to the cooking process performed under 120 °C. On the other hand, aliphatic GSLs appear to be more stable, with only a slight degradation has been observed under the same cooking conditions. The degradation became drastic for all GSL under canning conditions, whereas the process temperature exceeds 120 °C. The total amount of GSL has been reduced by over 70% under these harsh conditions. These observations are drawn from conclusions about the difference in thermal stabilities between aliphatic and indolic GSLs.

A study conducted by Song and Thornalley also reported the thermal degradation of GSL due to the domestic processing of *Brassica* vegetables, such as Brussel sprouts, broccoli, cauliflowers, and green cabbage [166]. Moreover, the effects of the cooking method, such as microwave, steam, and stir-fry, on GSL amounts of studied materials were investigated. The results showed that cooking by these cooking methods did not produce a significant loss of GSL, in contrast to boiling, which showed significant losses by leaching of GSL into cooking water at high temperatures [14]. Therefore, boiling *Brassica* should be avoided in order to preserve intact GSL in raw materials.

A recent study on the roasting process of rapeseed seed reported shows that industrial-scale post-harvest treatments, which are often necessary to produce higher quality oil-related products, also impact the GSL profile of plant materials [164]. Up to 29% of the original GSL amount in plant materials have been reduced during the roasting process. The results indicate that the industrial-scale roasting processes reduce the GSL amount of plant materials due to the thermal degradation, with up one-third of GSLs are degraded via thermal degradation.

Based on the information outlined above, we suggest that, with regards to downstream purposes, the selection of plant material should rely on the processing method. Although thermal treatments of plant materials, whereas the GSL content is often reduced, are beneficial for food and feed applications, these should be avoided in order to maintain the desired amount of GSL for non-food purposes. We highly recommend the review by Hanschen et al. [167] for further reading concerning the reactivity and stability of GSL and their breakdown products in food.

### 6.2. Degradation of Glucosinolates in Solution

The stability of GSL and desGSL from *Moringa oleifera* in solution was investigated with the presence and absence of buffer [40]. The GSL extracted from plant materials, either desulfated or intact, were dissolved in ultra-pure water and stored at room temperature or −20 °C. After nine days of storage, the GSL profile of the extracts was analyzed. The results showed that GSLs were stable at low temperatures with little isomeric conversion or degradation of GSLs having occurred. On the other hand, a GSL solution stored at room temperature showed conversion among acetylated GSL isomers. Furthermore, the degradation of GSLs has been reported to be up to 32% of the original total amount of GSL. At room temperature, buffered solutions of GSL appear to be more stable than those in water solution, with a reduction of 20% of the total amount of GSLs being recorded within nine days. There was no significant difference between unbuffered and buffered GSL stored at low temperatures. Based on this information, storing GSL in buffer solutions at low temperatures (at −20 °C, in preference) is suggested to safely conserve the original GSL profile in extract when GSL is required to be stored in solution instead of stable solid salt form.

## 7. Biological Activities of Glucosinolates

### 7.1. Mechanism of Myrosinase

GSL play an important role in the defense mechanism of *Brassica* plants. Upon tissue disruption, catabolites released by MYR-catalyzed hydrolysis are frequently responsible for the toxicity of the parent GSL, which, in contrast, are biologically inactive [168,169]. This mechanism of prevention against herbivory feeding suggested the main function of GSLs in plant defense systems [45].

The intact GSLs are stored separately from the thioglycosidase MYR. The latter catalyzes the hydrolysis of GSL upon plant tissue disruption. As described in Figure 1, an unstable aglucone moiety has been released alongside with the glucose during hydrolysis. The aglucone moiety then undergoes further transformation to yield a number of metabolites.

MYR belongs to the Glycosidase family (EC 3.2.1.). Although it catalyzes *S*-glycosylation, the deduced amino acid sequences of MYR reveal strong similarities with several *O*-glycosidases [170]. Furthermore, MYR displays a retaining mechanism that is similar to that of family 1-*O*-glycosidases [171]. In order to elucidate the mechanism of MYR, Burmeister et al. have studied the crystallographic structure of MYR [170,172].

**Figure 9:**
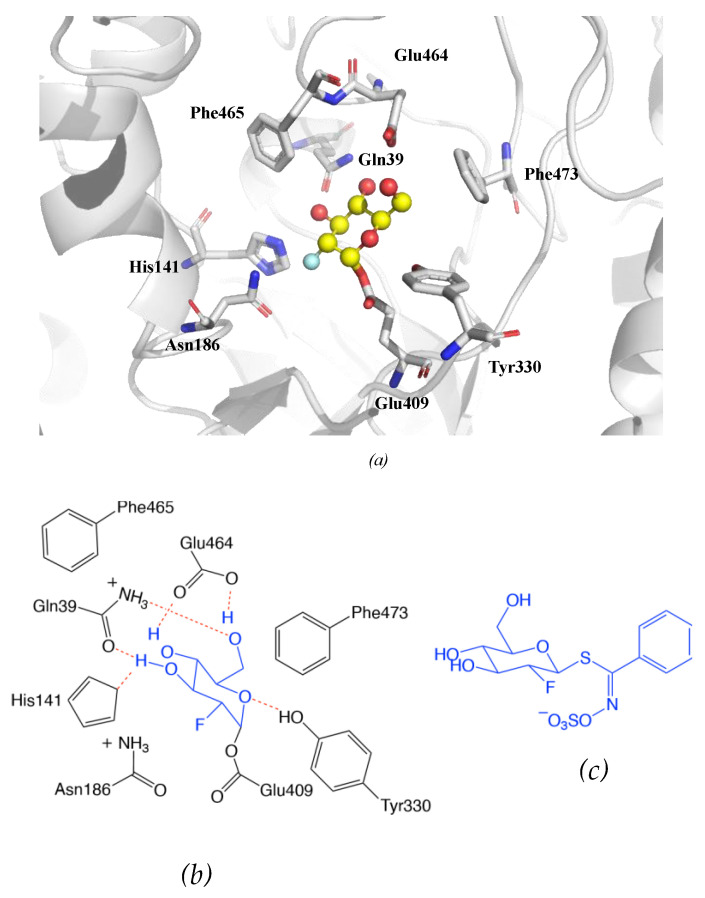
Overview of the active site of *Sinapis alba* Myrosinase showing interactions between residues and the 2-deoxy-2-fluoroglucosinolate (2FG) as substrate (Protein Data Bank accession number 1E70, resolution: 1.65 Å) [172]. Red dashed lines show hydrogen bonding interactions between the substrate and MYR residues within the active site. (**a**) Representation of the active site of *Sinapis alba* Myrosinase generated using PyMol. (**b**) Chemical structure representation of the MYR-2FG. (**c**) Structure of 2-deoxy-2-fluoroglucosinolate.

The crystallographic structure was generated by soaking the MYR crystals in 2-deoxy-2-fluoroglucosinolate (2FG) (Structure shown in Figure 9c). The results clearly showed that the 2-fluoroglucose moiety, released from the substrate upon myrosinase attack, is covalently bound to Glu409 within the active site (Figure 9a). The crystallization of 2FG-MYR complex confirmed MYRs as retaining glycosyl hydrolases.

**Figure 10:**
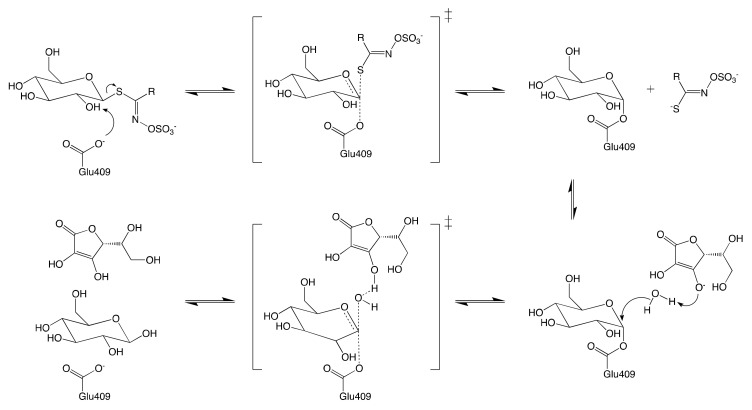
Schematic reaction mechanism of MYR in the presence of ascorbic acid.

Like most retaining glycosyl hydrolases, MYRs follow a conventional two-step mechanism: (1) the formation of covalent substrate-enzyme intermediate; and (2) the release of glucose via hydrolysis of the previously formed intermediate. The mechanism of glucose hydrolysis is described in Figure 10. The glycosylation begins with the introduction of GSL into the active site of MYR. The residue Glu406 then binds to the glucose moiety of the substrate at the anomeric position, releasing aglucone moiety.

Ascorbic acid was identified as a coenzyme of MYR for the first time by Ettlinger et al. [173]. Although it has been proved to be nonessential for the catalyzed hydrolysis of GSL [172], the presence of ascorbic acid enhances up to 400-fold the glycosylation of MYR [173]. The ultimate step consists in the release of both ascorbic acid and glucose from the active site to yield the enzyme in its native conformation.

#### 7.1.1. Hypothetical Recognition Role of Sulfate Group

Although represented as a characteristic of GSL, the sulfate group in the aglucone moiety exhibits an unclear function towards MYRs. Nonetheless, the distorted conformation of GSLs due to the interaction of the sulfate group with the amino acid side-chain of the myrosinase within its active site has been mentioned [172]. Based on these results, it was hypothesized that myrosinase recognizes glucosinolate substrates via the sulfate group.

Attempts to rationalize the recognitive function of the sulfate group have been conducted based on the feeding pattern of crucifer specialist insects. The investigation on *Plutella xylostella* larvae feeding pattern devised by Ratzka et al. suggested that the removal of the sulfate group renders GSLs invisible to MYR [174]. Furthermore, a number of articles have been published emphasizing the importance of the removal of the sulfate group of GSL which allows specialist insects to feed on crucifer plants [175,176,177].

These observations are strong proof supporting our hypothesis regarding the recognition role of the sulfate group within the defense system in crucifer plants. However, there is, to date, no further research article investigating the sulfate group of GSLs since the publication of the crystal structure of *Sinapis alba* MYR by Burmeister et al. [170,172]. Further investigation of the substrate recognition mechanism of MYRs will undoubtedly confirm the role of the sulfate group.

#### 7.1.2. Reconfiguration of Unstable Aglucone

**Figure 11:**
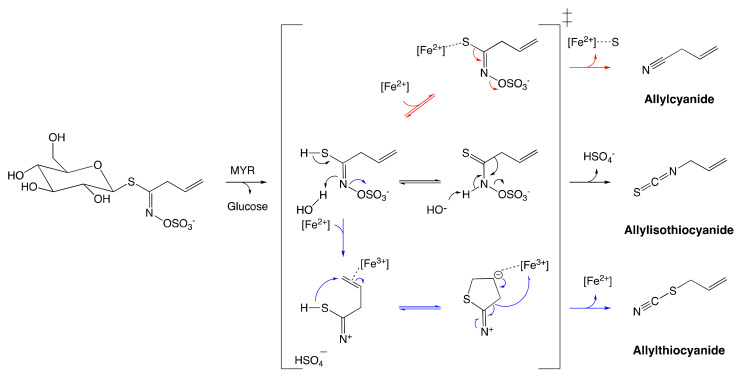
Reconfiguration of unstable allylglucosinolate aglucone upon myrosinase-catalyzed hydrolysis. The black arrow pathway shows the formation of allylisothiocyanates employing spontaneous Lossen arrangement. The Blue arrow pathway shows the formation of allylthiocyanate assisted by protein specifier. The red arrow pathway indicates the formation of allylcyanide assisted by protein specifier. The figure was adapted from Eisenschmidt-Bönn et al. [179].

As described previously, an unstable aglucone moiety of GSL is released alongside with a glucose unit upon MYR-catalyzed hydrolysis. A number of biologically active compounds are next obtained via the reconfiguration of unstable aglucone [14]. ITC, the most studied among GSL catabolites, is obtained via a spontaneous Lossen rearrangement of the corresponding aglucone under physiological conditions (Figure 11).

An additional range of bioactive non-ITC catabolites from MYR-catalyzed hydrolysis were also identified [14,178]. Sinigrin is the only known GSL that can form ITC alongside other products such as nitriles, epithionitriles, and thiocyanates (Figure 11). Their formation is regulated by the prerequisite allyl structure of the aglucone and the presence of protein specifiers [179]. It is noteworthy that these catabolites are as well obtained in low-yield in vitro at low pH in the presence of ferrous ions in spite of the absence of specifier-proteins [178]. These findings draw conclusions about the pH dependence of catabolite formation due to the reconfiguration of GSL aglucones [4].

### 7.2. Biological Activities of Glucosinolates and Their Catabolites

Negative effects of GSL on domestic animals have been documented by Tripathi and Mishra in their review [19]. These effects usually occur upon the assimilation of GSLs at high concentrations. Among relevant symptoms, reduction of feed intake, which causes growth depression, and induction iodine deficiency are often reported [180,181]. Moreover, high GSL diets eventually result in higher mortality in pigs, rats, and rabbits [19]. As such, an intake limit of GSL should be defined d in order to avoid the occurrence of unexpected negative effects.

To the best of our knowledge, there is no clear evidence in the literature indicating the negative effect of GSL on human health upon assimilation. In contrast, GSL catabolites such as ITC and nitrile have been proved to provide attractive therapeutic effects such as the induction of phase II enzymes [20]. The augmentation of tissue levels of the phase II detoxification enzymes is associated with decreased susceptibility to chemical carcinogenesis [182]. In their study, Munday and Munday observed an increase in the phase II detoxification enzymes, such as quinone reductase and glutathione *S*-transferase in rat tissues by daily oral-assimilating of different ITC compounds [20]. The authors, therefore, suggested that chemoprotective effects are common in ITC.

GSL catabolites are potent inhibitors of bacterial activity [8]. Although intact GSL was usually bio-inactive [19], allyl ITCs exhibit antimicrobial activities. By studying the effect of allyl ITCs on *Staphylococcus aureus*, a methicillin-resistant bacterium that causes purulent skin and soft tissue infections, Dias et al. concluded that these molecules issued from catalyzed-hydrolysis GSL possess strong antimicrobial activity against these specific bacteria [183].

Biofumigation is a process where plants are used as natural “pesticides” to reduce soil-borne pests and pathogens. Biofumigation properties of GSL and their breakdown products have been investigated by Haschen et al. [184]. In their study, the cultivation of *Brassica juncea* produced a significant amount of GSL and their hydrolysis products, such as ITC and nitrile, and released them into the cultivating soil. Consequently, the inhibition of bacterial community growth that cannot support the effects of breakdown products of GSLs has been observed. These results confirmed the fumigation properties of GSLs and their breakdown products.

In other circumstances, GSLs are catalytically hydrolyzed in vivo by supplementary proteins known as specifier proteins [185]. These latter promote the formation of non-ITC catabolites such as nitriles, epithionitriles, and thiocyanates, of which biological roles have been reviewed [179]. The coexistence of specifier proteins, along with MYR suggests the adaptation of the plant to circumvent the presence of natural enemies. For instance, favoring the production of simple nitriles over ITC upon herbivore damage enables better defense of *A. thaliana* against the specialist herbivore [186].

## 8. Conclusions

Recently, GSL and its breakdown products have been studied extensively with regard to their therapeutic and agricultural benefits. The diversity of side-chains, with over 130 GSL structures identified and validated to date, alongside with their abundant presence in *Brassica* plants, make these metabolites of great interest for natural product chemistry, biochemistry, and biology.

Despite the advanced development of synthetic approaches, extraction of naturally occurring GSL from corresponding plant materials remains the method of choice to obtain these molecules of interest. On the other hand, extraction approaches have several drawbacks that must be overcome before they can be employed routinely at the industrial scale. The high hydrophilicity of GSLs restrains their separation from the aqueous extract by conventional extraction methods. The purification and characterization of individual GSLs often require advanced chromatography techniques, which are criticized for being costly, time- and labor-consuming. As a result, designing and optimizing more straightforward, accessible, and sustainable extraction methods for GSLs remains a challenge.

## Figures and Tables

**Table 1:** Occurrence of GSL in plants of order *Brassicales*. GSL concentration is expressed as a minimum–maximum in µmol/g of dry material.

Family	Species	Tissue	GSL Content	Reference
*Brassicaceae*	*Camelina sativa*	Seed	15.8–19.4	[30]
	*Camelina rumelica* subsp. *rumelica*	Seed	18.6–21.7	
	*Camelina macrocarpa*	Seed	8.0–19.1	
	*Brassica napus*	Leaf	0.6–6.9	[22,31,32]
		Seed	10.8–57.9	
	*Brassica carinata* A Braun	Seed	35–170	[26,27]
	*Brassica juncea*	Leaf	4.3–129.9	[33]
		Seed	15.7–127.6	
	*Brassica oleracea* L. var *capitata*	Leaf	2.3–11.5	[34]
	*Brassica oleracea* L. var *italica*	Floret	8.2–19.5	[35]
	*Brassica oleracea* L. convar *capitata* var *alba*	Petiole	0.5–31.7	[36]
	*Brassica rapa*	Leaf	17.3	[31]
		Seed	39.4–81.3	
	*Arabidopsis thaliana*	Leaf	5.0–30.7	[37]
	*Raphannus sativus* L.	Root	1.0–145.5	[29,38]
*Moringacea*	*Moringa oleifera* Lam.	Leaf	4.7–217	[28,39]
		Seed	112–354.4	[40,41]
	*Moringa stenopetala* L.	Leaf	33.9–59.4	[42,43]
		Seed	256–282	

**Table 2:** Classification of GSL structural examples validated by Blazevic et al. [4]. Index A, B, and C indicate aliphatic, aromatic, and indolic GSLs, respectively.

No	Class	Index	Semi Systematic Name	Trivial Name	Characterization Methods	Reference
1	Ala	A	Methyl GSL	Glucocapparin	MS, NMR of GSL; MS of desGSL	[47,48]
2	Val	A	1-Methylethyl GSL	Glucoputranjivin	UV, IR, MS, NMR of GSL	[49]
3	Val	A	(1*R*)-Methyl-2-hydroxyethyl GSL	Glucosisymbrin	MS, NMR of desGSL	[50,51]
4	Val	B	(1*R*)-2-Bezoyloxt-1-methylethyl GSL	Glucobenzosisymbrin	UV, IR of ITC	[52]
5	Glu	A	3-Carboxypropyl GSL		Deducted from ITC structure	[53]
6	Glu	A	3-Methoxycarbonyl- propyl GSL	Glucoerypestrin	Partial NMR of GSL	[54]
7	?	A	Ethyl GSL	Glucolepidiin	Thiourea-type, IR compared to GSL structure	[55]
8	?	A	*n*-Butyl GSL		Thiourea-type method compared to GSL	[56,57]
					and MS from ITC	
9	?	A	*n*-Pentyl GSL		MS of ITC	[58]
10	?	A	*n*-Hexyl GSL		MS of ITC	[57]
11	?	A	4-Oxoheptyl GSL	Glucocapangulin	Deducted from IR and 5-oxooctanoic acid	[59]
12	?	A	5-Oxoheptyl GSL	Gluconorcappasalin	Thiourea-type, IR compared to GSL;	[60]
					MS from ITC	
13	?	A	5-Oxooctyl GSL	Glucocappasalin	UV, IR of GSL and desGSL; partial NMR of desGSL	[61]
14	?	A	4,5,6,7-Tetrahydroxydecyl GSL		UV, IR, NMR of ITC	[62]
15	?	B	Phenyl GSL		MS of GSL	[57]
16	?	B	2-(4-Methoxyphenyl)-2,2-dimethyl ethyl GSL		IR, MS, NMR of ITC	[63]
17	Leu	A	2-Methylpropyl GSL		MS, NMR of GSL and desGSL	[50,64]
18	Leu	A	2-Hydroxy-2-methylpropyl GSL	Glucoconringiin	MS, NMR	[65]
19	Leu	A	3-Methylbutyl GSL		MS of ITC	[58]
20	Leu	A	3-Methylbut-3-eyl GSL		IR, MS, NMR of	[66]
					ITC	
21	Leu	A	4-Methylpentyl GSL		MS of ITC	[67]
22	Ile	A	(1*S*)-1-Methylpropyl GSL	Glucocochlearin	MS, NMR of GSL and desGSL	[50,68]
23	Ile	A	(1*R*)-1-(Hydroxymethyl)-propyl GSL	Glucosisaustricin	MS, NMR of desGSL	[50]
24	Ile	B	(1*R*)-1-(Benzoyloxymethyl)-propylGSL	Glucobenzsisaustricin	Thiourease-type, IR compared to GSL	[69]
25	Ile	A	(2*S*)-2-Methylbutyl GSL	Glucojiaputin	UV, IR, MS, NMR of GSL and des GSL	[49,50]
26	Ile	A	(2*S*)-2-Hydroxy-2-methylbutyl GSL	Glucocleomin	NMR of desGSL	[51]
27	Ile	A	3-Methylpentyl GSL		UV, IR, MS, NMR of GSL; MS, NMR of desGSL	[49,50]
28	Ile	A	3-(Hydroxymethyl)pentyl GSL		NMR of GSL	[70]
29	Ile	A	2-Hydroxy-3-methylpenyl GSL		MS, NMR of desGSL	[50,70]
30	Trp	C	4-Methoxyindol-3-yl GSL	Glucorapassicin A	UV, IR, MS, NMR of synthesized GSL	[71]
31	Trp	C	Indol-3-ymethyl GSL	Glucobrassicin	UV, IR, MS, NMR of GSL and desGSL	[49,50]
32	Trp	C	1-Hydroxyindol-3ylmethyl GSL		MS of GSL; UV, MS of desGSL	[72]
33	Trp	C	4-Hydroxyindol-3-ylmethyl GSL	4-Hydroxy-glucobrassicin	MS of GSL; UV, MS, NMR of desGSL	[72,73,74]
34	Trp	C	4-Methoxyindol-3-ylmethyl GSL	4-Methoxy-glucobrassicin	UV, MS, MS, NMR of GSL and desGSL	[49,74]
35	Trp	C	1-Methoxyindol-3-ylmethyl GSL	Neoglucobrassicin	UV, IR MS, NMR of GSL; MS, NMR of desGSL	[49,50,72]
36	Trp	C	1,4-Dimethoxyindol-3-ymethyl GSL	1,4-Dimethoxy-glucobrassicin	UV, MS, NMR ofdesGSL	[50,70]
37	Trp	C	1-Acetylindol-3-ymethyl GSL	*N*-Acetyl-glucobrassicin	MS of desGSL	[75]
38	Trp	C	1-Sulfoindol-3-ylmethyl GSL	*N*-Sulfo-glucobrassicin	UV, IR, MS, NMR of GSL	[65,76]
39	Trp	C	6′-Isoferuloylindol-3-ylmethyl GSL	6′-Isoferuloyl-glucobrassicin	MS of GSL; UV, MS, NMR of desGSL	[77,78]
40	Phe	B	Benzyl GSL	Glucotropaeolin	MS, NMR of GSL; UV, MS, NMR of desGSL	[51,79,80]
41	Phe	B	3-Hydroxybenzyl GSL	Glucolepigramin	MS of GSL; MS, NMR of desGSL	[65,81]
42	Phe	B	3-Methoxybenzyl GSL	Glucolimnanthin	MS, NMR of GSL; UV, MS, NMR of desGSL	[51,82]
43	Phe/Trp	B	4-Hydroxybenzyl GSL	Glucosinalbin	UV, MS, NMR of GSL and desGSL	[52,78,83]
44	Phe/Trp	B	4-Methoxybenzyl GSL	Glucoaubrietin	MS of GSL; UV,	[50,65,84]
					MS, NMR of	
					desGSL	
45	Phe/Trp	B	3,4-Dihydroxybenzyl GSL	Glucomatronalin	MS of GSL	[65]
46	Phe/Tyr	B	4-Hydroxy-3-methoxybenzyl GSL	3-Methoxysinalbin	UV, MS, NMR of desGSL	[81]
47	Phe/Tyr	B	3-Hydroxy-4-methoxybenzyl GSL	Glucobretschneiderin	UV, IR, MS, NMR of GSL	[85]
48	Phe/Tyr	B	3,4-Dimethoxybenzyl GSL		UV, MS, NMR of	[81]
					desGSL	
49	Phe/Tyr	B	4-Hydroxy-3,5-dimethoxybenzyl GSL	3,5-Dimethoxy-sinalbin	UV, MS, NMR of desGSL	[81]
50	Phe/Tyr	B	3,4,5-Trimethoxybenzyl GSL		MS of GSL; UV, MS, NMR of desGSL	[80,81]
51	Phe	B	2-Phenylethyl GSL	Gluconasturtiin	NMR of GSL; UV, MS, NMR of desGSL	[51,65]
52	Phe	B	(2*S*)-2-hydroxy-2-phenylethyl GSL	Glucobarbarin	MS, NMR of GSL and desGSL	[50,65,74]
53	Phe	B	(2*R*)-2-Hydroxy-2-phenylethyl GSL	Epiglucobarbarin	MS, NMR of GSL and des GSL	[65,77,86]
54	Phe	B	2-(3-Hydroxy-phenyl)ethyl GSL		UV, MS, NMR of desGSL	[86]
55	Phe	B	2-(4-Hydroxy-phenyl)ethyl GSL	Homosinalbin	MS, NMR of GSL; UV, MS, NMR of desGSL	[65,87]
56	Phe	B	(2*R*)-2-Hydroxy-2-	*m*-Hydroxy-epiglucobarbarin	UV, MS, NMR of GSL and desGSL	[18]
			(3-hydroxyphenyl)ethyl GSL			
57	Phe	B	3-Phenylpropyl GSL		MS of ITC	[88]
58	Phe	B	4-Phenylbutyl GSL		MS of ITC	[88]
59	Phe	B	5-Phenylpentyl GSL	Glucoarmoracin	MS of ITC	[88]
60	Phe/Tyr	B	2-(4-Methoxy-phenyl)ethyl GSL		NMR of GSL, MS, NMR of desGSL	[65,70,84]
61	Phe/Tyr	B	(2*R*)-2-Hydroxy-2-(4-hydroxyphenyl)ethyl GSL	*p*-Hydroxy-epiglucobarbarin	MS, NMR of GSL; UV, MS, NMR of desGSL	[70,89]
62	Phe/Tyr	B	(2*S*)-2-Hydroxy-2(4-hydroxyphenyl)ethyl GSL	*p*-Hydroxy-glucobarbarin	UV, MS, NMR of desGSL	[89]
63	Phe/Tyr	B	(2*R*)-2-Hydroxy-2(4-methoxyphenyl)ethyl GSL		MS, NMR of GSL	[90]
64	Phe	B	2-(*α*-l-Rhamnopyranosyloxy)-benzyl GSL		MS of GSL and desGSL	[65,87]
65	Phe	B	4-(4′-*O*-Acetyl-*α*-l- 4- rhamnopyranosyloxy)-benzyl GSL	4-Acetyl-glucomoringin	MS of GSL and ITC	[43,91]
66	Phe	B	2-(*α*-l-Arabinopyranosyloxy)-2phenylethyl GSL		NMR of GSL	[92]
67	Phe	B	6′-Isoferuloyl-2-phenylethyl GSL	6′-Isoferuloyl-gluconasturtiin	MS of GSL, UV, MS NMR of desGSL	[77,78]
68	Phe	B	6′-Isoferuloyl-(2*R*)-2-hydroxy-2phenylethyl GSL	6′-Isoferuloyl-epiglucobarbarin	MS, NMR of GSL; UV, MS, NMR of desGSL	[78]
69	Phe	B	6′-Isoferuloyl-(2*S*)-2-hydroxy-2phenylethyl GSL	6′-Isoferuloyl-glucobarbarin	MS, NMR of GSL; UV, MS, NMR of desGSL	[78]
70	Phe/Tyr	B	6′-Isoferuloyl-(*R*)-2-hydroxy-2(4-hydroxyphenyl)ethyl GSL		MS of GSL; UV, MS NMR of desGSL	[78]
71	Phe/Tyr	B	4-(*α*-l-Rhamnopyranosyloxy)-benzyl GSL	Glucomorinigin	MS, NMR of GSL and desGSL	[51,93,94,95]
72	Met	A	3-(Methylsulfanyl)propyl GSL	Glucoibervirin	MS, NMR of GSL	[96]
73	Met	A	4-Oxoheptyl GSL	Glucocapangulin	Deduction from IR, 5-oxooctanoic acid	[59]
74	Met	A	4-(Methylsulfanyl)butyl GSL	Glucoerucin	UV, IR, MS NMR of GSL	[51,80]
75	Met	A	5-(Methylsulfanyl)pentyl GSL	Glucoberteroin	UV, IR, MS, NMR of GSL; UV, MS, NMR of desGSL	[97,98,99]
76	Met	A	6-(Methylsulfanyl)heptyl GSL		UV, IR, MS, NMR of GSL	[97,98]
77	Met	A	6-(Methylsulfanyl)hexyl GSL	Glucolesquerellin	UV, IR, MS, NMR of GSL	[97,98]
78	Met	A	8-(Methylsulfanyl)-3-oxooctyl GSL		MS of GSL; MS, NMR of des GSL	[50,65]
79	Met	A	9-(Methylsulfanyl)nonyl GSL		MS of GSL	[65]
80	Met	A	10 -(Methylsulfanyl)decyl GSL		MS of ITC	[100]
81	Met	A	2-Methylsulfinylethyl GSL		UV, MS, NMR of	[101]
					desGSL	
82	Met	A	(*R*)-11-(Methylsulfinyl)-propyl glucosinolate	Glucoiberin	MS, NMR, X-Ray of GSL; UV, MS, NMR of desGSL	[51,80,102]
83	Met	A	(*R/S*)-4-(Methylsulfinyl)-butyl glucosinolate	Glucoraphanin	MS, NMR of GSL, UV, MS NMR of desGSL	[51,80,103]
84	Met	A	(*R/S*)-5-(Methylsulfinyl)pentyl GSL	Glucoalyssin	MS, NMR of GSL; MS of desGSL	[80,104]
85	Met	A	(*R/S*)-6-(Methylsulfinyl)-hexyl GSL	Glucohesperin	UV, IR, MS, NMR of GSL	[80,97,98]
86	Met	A	(*R/S*)-7-(Methylsulfinyl)-heptyl GSL		NMR of GSL; MS, NMR of desGSL	[50,105]
87	Met	A	(*R/S*)-8-(Methylsulfinyl)-octyl GSL	Glucohirsutin	UV, IR, MS, NMR of GSL; MS, NMR of desGSL	[50,98]
88	Met	A	(*R/S*)-9-(Methylsulfinyl)-nonyl GSL	Glucoarabin	UV, IR, MS, NMR of GSL; MS, NMR of desGSL	[98,106]
89	Met	A	(*R/S*)-10-(Methylsulfinyl)decyl GSL	Glucocamelinin	MS, NMR of GSL; MS of desGSL	[50,107]
90	Met	A	(*R/S*)-11-(Methylsulfinyl)undecyl GSL		MS of GSL	[107]
91	Met	A	3-(Methylsulfonyl)-propyl GSL	Glucocheirolin	MS of GSL; NMR of desGSL	[48,108]
92	Met	A	4-(Methylsulfonyl)butyl GSL	Glucoerysolin	MS of GSL; MS, NMR of desGSL	[65,84,106]
93	Met	A	6-(Methylsulfonyl)hexyl GSL		MS of GSL	[65]
94	Met	A	8-(Methylsulfonyl)octyl GSL		UV, IR, MS, NMR of GSL; MS, NMR of desGSL	[50,106,109]
95	Met	A	9-(Methylsulfonyl)nonyl GSL		UV, IR, MS, NMR of GSL; MS, NMR of desGSL	[50,106,109]
96	Met	A	10-(Methylsulfonyl)decyl GSL		MS, NMR of desGSL	[84,106]
97	Met	A	(3*E*)-4-(Methylsulfanyl)-but-3-enyl GSL		IR, MS, NMR of GSL; NMR of desGSL	[51,80]
98	Met	A	(*R/S*,3*E*)-4-(Methylsulfinyl)-but-3-enyl GSL	Glucoraphenin	MS, NMR of GSL; UV, NMR of desGSL	[51,80,110]
99	Met	A	3-Hydroxy-5-(methylsulfinyl)pentyl GSL		Deducted from tetrahydro-1,3-oxazine-2-thione	[111]
100	Met	A	3-Hydroxy-5-(methylsulfony)pentyl GSL		UV, IR, MS, NMR of ITC	[111]
101	Met	A	3-Hydroxy-6-(methylsulfanyl)hexyl GSL		Deducted from tetrahydro-1,3-oxazine-2-thione	[112]
102	Met	A	3-Hydroxy-6-(methylsufinyl)hexyl GSL		Deducted from ITC	[112]
103	Met	A	3-Hydroxy-5-(methylsulfinyl)pentyl GSL		Deducted from tetrahydro-1,3oxazine-2-thione and ITC	[112]
104	Met	A	8-(Methylsulfanyl)-3-oxooctyl GSL		Deducted from ITC	[113]
105	Met	A	(*R/S*)-8-(Methylsulfinyl)-3-oxooctyl GSL		Deducted from ITC	[113]
106	Met	A	4-Mercaptobutyl GSL		MS, NMR of GSL	[114,115]
107	Met	A	(*R*)-4-(Cystein-*S*-yl)butyl GSL	Glucorucolamine	MS, NMR of desGSL	[116]
108	Met	A	Dimeric 4-mercaptobutyl GSL		MS, NMR of GSL; MS of desGSL	[114]
109	Met	A	4-(*β*-d-Glucopyranosyl- disulfanyl)-butyl GSL	Diglucothiobeinin	MS of GSL; MS, NMR of desGSL	[117,118]
110	Met	A	6′-Benzoyl-4(methylsulfanyl)butyl GSL	6′-Benzoyl-glucoerucin	UV, MS, NMR of desGSL	[101]
111	Met	A	6′-Benzoyl-4(methylsulfinyl)butyl- GSL	6′-Benzoyl -glucopharanin	UV, MS, NMR of desGSL	[101]
112	Met	A	(*R/S*, *3E*)-6′-Sinapoyl-4-(methylsulfinyl)but-3-enyl GSL	6′-Sinapoyl-glucoraphenin	UV, IR, MS, NMR of desGSL	[119]
113	Se-Met	A	3-(Methylseleno)propyl GSL		Comparing MS with natural S-analog	[120]
114	Se-Met	A	4-(Methylseleno)butyl GSL		Comparing MS with natural S-analog	[120]
115	Se-Met	A	5-(Methylseleno)pentyl GSL		Comparing MS with natural S-analog	[120]
116	Met	A	Allyl glucosinolate	Sinigrin	MS, NMR, X-Ray of GSL; UV, MS, NMR of desGSL	[51,65]
117	Met	A	But-3-enyl GSL	Gluconapin	MS, NMR of GSL; UV, MS, NMR of desGSL	[50,51,80,104]
118	Met	A	Pent-4-enyl GSL	Glucobrassicanapin	MS of GSL; MS, NMR of desGSL	[50,104]
119	Met	A	(*2S*)-2-Hydroxypent-4-enyl GSL	Gluconapoleiferin	MS of GSL	[73]
120	Met	A	(*2R*)-2-Hydroxybut-3-enyl GSL	Progoitrin	MS, NMR of GSL; UV, MS, NMR of desGSL	[50,51,79,80,84]
121	Met	A	(*2S*)-2-Hydroxybut-3-enyl GSL	Epiprogoitrin	MS, NMR of GSL; UV, MS, NMR of desGSL	[50,51,73,79]
122	Met	C	2′,3′-Dihydro-2′-oxoindol-3′-ylacetate ester at 2-OH of (*R*)-2-hydroxbut-3-enyl GSL	Glucoisatisin	UV, MS, NMR of GSL	[121,122]
123	Met	C	2′,3′-Dihydro-2′-oxoindol-3′-ylacetate of ester at 2-OH of (*S*)-2-hydroxbut-3-enyl GSL	Epiglucoisatisin	UV, MS, NMR of GSL	[121,122]
124	Met	C	2′,3′-Dihydro-3′-hydroxy-2′-oxoindol-3′-ylacetate ester at 2-OH of (*R*)-2-hydroxybut-3-enyl GSL	(*S*)-3′-Hydroxy-glucoisasitin	UV, MS, NMR of GSL	[121]
125	Met	C	2′,3′-Dihydro-3′-hydroxy-2′-oxoindol-3′-ylacetate ester at 2-OH of (*S*)-2-hydroxybut-3-enyl GSL	(*S*)-3′-Hydroxy-epiglucoisasitin	UV, MS, NMR of GSL	[121]
126	Met	B	(*2S*)-2-Benzoyloxybut-3-enyl GSL	2-*O*-Benzoyl-epiprogoitrin	MS of desGSL	[101]
127	Met	A	2-Hydroxyethyl GSL		NMR of GSL	[123]
128	Met	A	3-Hydroxypropyl GSL		MS, NMR of ITC	[124]
129	Met	A	4-Hydroxylbutyl GSL		MS of GSL	[125]
130	Met	A	3-Hydroxylbutyl GSL		Deducted from tetrahydro-1,3oxazine-2-thione	[56]
131	Met	B	2-(Benzoyloxy)ethyl GSL		MS of GSL	[125]
132	Met	B	3-(Benzoyloxy)propyl GSL	Glucomalcolmiin	MS of GSL; UV, MS of desGSL	[101,125,126]
133	Met	B	4-(Benzoyloxy)butyl GSL		MS of GSL; UV, MS, NMR of desGSL	[101,125]
134	Met	B	5-(Benzoyloxy)pentyl GSL		MS, NMR of desGSL	[101]
135	Met	B	6-(Benzoyloxy)hexyl GSL		Deducted from ITC	[126]
136	Met	B	3-Sinapoyloxypropyl GSL		MS, NMR of desGSL	[127]
137	Met	B	6′-Benzoyl-4-benzoyloxybutyl GSL		UV, MS, NMR of desGSL	[101]

desGSL: desfulfated Glucosinolates; IR: Infra-red; ITC: Isothiocyanates; MS: Mass Spectrometry; NMR: Nuclear Magnetic Resonance; Index: ?: Uncertain precursor.

## Abbreviations

desGSL: desfulfated Glucosinolates; GSL: Glucosinolates; IR: Infra-red; ITC: Isothiocyanates; MS: Mass Spectrometry; MYR: Myrosinase; NMR: Nuclear Magnetic Resonance.
